# Competence Classification of Cumulus and Granulosa Cell Transcriptome in Embryos Matched by Morphology and Female Age

**DOI:** 10.1371/journal.pone.0153562

**Published:** 2016-04-29

**Authors:** Rehannah Borup, Lea Langhoff Thuesen, Claus Yding Andersen, Anders Nyboe-Andersen, Søren Ziebe, Ole Winther, Marie Louise Grøndahl

**Affiliations:** 1 Center for Genomic Medicine, University Hospital of Copenhagen, Rigshospitalet, Copenhagen, Denmark; 2 Fertility Clinic, University Hospital of Copenhagen, Rigshospitalet, Copenhagen, Denmark; 3 Laboratory of Reproductive Biology, University Hospital of Copenhagen, Rigshospitalet, Copenhagen, Denmark; 4 Bioinformatics Center, Department of Biology and Biotech Research and Innovation Centre, University of Copenhagen, Copenhagen, Denmark; 5 Fertility Clinic, University Hospital of Copenhagen, Herlev Hospital, Copenhagen, Denmark; Inner Mongolia University, CHINA

## Abstract

**Objective:**

By focussing on differences in the mural granulosa cell (MGC) and cumulus cell (CC) transcriptomes from follicles resulting in competent (live birth) and non-competent (no pregnancy) oocytes the study aims on defining a competence classifier expression profile in the two cellular compartments. Design: A case-control study. Setting: University based facilities for clinical services and research. Patients: MGC and CC samples from 60 women undergoing IVF treatment following the long GnRH-agonist protocol were collected. Samples from 16 oocytes where live birth was achieved and 16 age- and embryo morphology matched incompetent oocytes were included in the study.

**Methods:**

MGC and CC were isolated immediately after oocyte retrieval. From the 16 competent and non-competent follicles, mRNA was extracted and expression profile generated on the Human Gene 1.0 ST Affymetrix array. Live birth prediction analysis using machine learning algorithms (support vector machines) with performance estimation by leave-one-out cross validation and independent validation on an external data set.

**Results:**

We defined a signature of 30 genes expressed in CC predictive of live birth. This live birth prediction model had an accuracy of 81%, a sensitivity of 0.83, a specificity of 0.80, a positive predictive value of 0.77, and a negative predictive value of 0.86. Receiver operating characteristic analysis found an area under the curve of 0.86, significantly greater than random chance. When applied on 3 external data sets with the end-point outcome measure of blastocyst formation, the signature resulted in 62%, 75% and 88% accuracy, respectively. The genes in the classifier are primarily connected to apoptosis and involvement in formation of extracellular matrix. We were not able to define a robust MGC classifier signature that could classify live birth with accuracy above random chance level.

**Conclusion:**

We have developed a cumulus cell classifier, which showed a promising performance on external data. This suggests that the gene signature at least partly include genes that relates to competence in the developing blastocyst.

## Introduction

In order to improve the efficacy of in vitro fertilisation treatment, there is ongoing research to define good non-invasive embryo markers to increase the implantation rate from an average of around 25% achieved with the existing morpho-kinetic markers [1].

The developmental competence of the mammalian oocyte is achieved during folliculogenesis, where cross-talk between oocyte and somatic cells ensures oocyte growth and maturation and the subsequent potential to sustain fertilisation and produce viable embryos [2]. Thus, several attempts have been made to identify oocyte competence markers in the various follicle compartments.

Specific functions of the somatic cells from the pre-ovulatory follicles just prior to ovulation (e.g. progesterone and oestrogen production from the cumulus-oocyte-complex (COC) [3] and expression levels of specific genes in the mural granulosa cells (MGC) and cumulus cells (CC) have been shown to reflect the developmental competence of the corresponding oocyte. Several authors have used a single gene approach by analyzing few genes by RT-PCR [4–7], while others have used global gene expression profiling analysis to find differences in somatic cell gene expression between cells connected to competent and non-competent oocytes [8–16]. These studies represent different end-points for competence that vary from oocyte maturational stage to the cleavage stage embryo morphology, positive hCG, ongoing pregnancy and finally birth of a healthy child. The difference in competence measures may partly explain the predominant lack of overlap in suggested competence marker genes, as may the difference in isolation technique, and the in vitro exposure time before isolation of cells.

Taking the complexity of the preovulatory follicle into account, the aim of identifying single marker genes seems to be less robust than a marker based on the scenario of multi interacting genes, which may be identified by the high trough put techniques such as the expression array. Therefore the present study focus on differences in the MGC and CC transcriptomes between oocytes from competent and non-competent follicles and the aim is to develop classifier profiles in the two cellular compartments in order to be able to select the embryos with the best potential to result in life offspring. To approach the scenario for selecting a competent embryo for transfer in a cohort of embryos, a case control design was chosen matching women’s age and embryo morphology.

Thus, in the present study, gene expression profiling of corresponding CC and MGC of individual follicles obtained from women undergoing IVF with elective single embryo Day 3 transfer were associated to no pregnancy after IVF or life birth of a healthy baby.

## Material and Methods

### Study population and treatment protocol

The CC and MGC were collected at the Fertility Clinic, Copenhagen University Hospital, Rigshospitalet, Denmark between February 2009 and June 2010 as a part of a prospective, randomised study (0 IU hCG; 50 IU hCG, 100 IU hCG and 150 IU hCG daily during controlled ovarian stimulation (COS)) [17]. The Danish National Committee on Biomedical Research Ethics (HB-2008-146) and The Danish Medicines Agency (2612–3928, EudraCT number 2008-008355-42) approved the study. All participants provided both written and verbal informed consent. The consent was documented with participant’s signature and the consent form was approved by the Ethics Committes.

A total of 60 women, scheduled for IVF, aged 25–37 years and with BMI >18 and < 30 kg/m^2^, regular menstrual cycle, early follicular phase serum FSH levels within 1–12 IU/l and early follicular phase total antral follicle (2–10mm) count ≥ 6 donated CC and MGC. The patients underwent controlled ovarian stimulation in a GnRH agonist protocol as described in [17]. Oocyte retrieval was performed 36 h (±2 h) after hCG administration. Single embryo transfer was performed Day 3 and 16 pregnancies resulted in live births. All clinical (week 7) and ongoing pregnancies (week 10–12) were confirmed with an ultrasound scan and followed up to delivery. The sixteen CC and corresponding MGC representing the competent oocytes and follicles giving rise to the babies were included in the study. The competent oocytes were evenly distributed in the 4 study groups [17]. To represent incompetent oocytes, another 16 CC and corresponding MGC were chosen among the samples with follicle cells connected to the oocytes that gave rise to an embryo that failed to implant. These samples were chosen to match the competent oocytes in relation to COS treatment, female age and embryo morphology.

For demographic data t-test and chi-squared tests were used and p<0.05 considered significant.

### Cumulus and granulosa cell isolation

Follicular fluids from all follicles were collected individually in tubes (Nunc Centrifuge 11 ml, Nunc, Denmark). When an oocyte was obtained, the number of the follicle was recorded and the oocytes were numbered consecutively. Thus, each oocyte could be linked to an individual follicle and hence the correspondent MGC. Immediately after isolation of the COC, the MGC were isolated from the follicular fluid by centrifugation at 400 g in 8 min at room temperature. The pellet was re-suspended in 1 ml HEPES-buffered solution (Sydney IVF Follicle Flush Buffer, COOK Medical, Australia) and placed on a density gradient column (Histopaque 1077, Sigma-Aldirch, St-Louis, USA) and centrifuged at 400 g for 30 minutes. After centrifugation, the inter-phase containing the MGC was aspirated and the MGC washed twice by mixing with1 ml of the buffer solution followed by centrifugation at 400 g for 8 minutes at room temperature. After the second wash, pellet was re-suspended in 500 μl in a cryo-vial (CryoVial, 2 ml Greiner bio-one, Germany) and stored at -80°C until RNA extraction.

Within 1 hour after the oocyte retrieval a part of the cumulus mass was mechanically removed (18G needles) from the COC and transferred to a cryo-vial (CryoVial, 2 ml Greiner bio-one, Germany) with as little fluid as possible and stored at -80°C until RNA extraction.

### Embryo Evaluation

IVF insemination was done with standard IVF procedure at 3 h (±1 h) after oocyte retrieval. All oocytes were followed individually and zygote and embryo quality assessed at 20 h (±1 h), 28 h (±1 h), 44 h (±1 h) and 68 h (±1 h) after oocyte retrieval. The embryo quality evaluation consisted of assessment of cell number and three parameters of embryo morphology: degree of fragmentation, cell stage specific blastomere uniformity and multinucleation (ALPHA and ESHRE consensus parameters, 2011).

### RNA purification

RNA from individual samples was purified using Arcturus PicoPure RNA Isolation kit (Applied Biosystems®, Naerum, Denmark) according to manufactures instructions but with small modifications. In short: 100 μl extraction buffer was used for initial lysis of each sample, except for samples were sample volume excided 70 μl. For these samples 2 x sample volume of extraction buffer was added. Following lysis and centrifugation, the supernatant was transferred to a 2 ml Eppendorf tube from the kit (Arcturus PicoPure RNA Isolation kit, Applied Biosystems®, Naerum, Denmark) where it was mixed 1:1 with 70% ethanol. For some MGC samples with large volume, several transfers and spins were needed to collect all the RNA in the spin tubes. In the end, the RNA was extracted in 12 μl nuclease free water, giving a ~10 μl end volume. The integrity of the RNA was measured using bioanalyzer pico chip (RNA 6000 Pico Assay for 2100 bioanalyzer, Agilent Technologies) and only samples with a RNA integrity number (RIN) above 7 as analyzed by the 2100 bioanalyzer (Agilent Technologies, Glostrup, Denmark) were included in the analysis.

### Microarray analysis

RNA was amplified and labeled using a pico amplification kit according to manufactures instructions. In short, approximately half of the total RNA from each sample (5 μl of 10 μl) was amplified using the Ovation Pico WTA v.2 RNA Amplification System from NuGEN® Inc. (NuGEN®, San Carlos, CA, USA) and biotin labeling was performed with the Encore Biotin Module (NuGEN®). The labeled samples were hybridized to the Human Gene 1.0 ST GeneChip array (Affymetrix, Santa Clara, CA, USA). The arrays were washed and stained with phycoerytrin conjugated streptavidin (SAPE) using the Affymetrix Fluidics Station® 450, and the arrays were scanned in the Affymetrix GeneArray® 3000 7G scanner to generate fluorescent images, as described in the Affymetrix GeneChip® protocol. Cell intensity files (CEL files) were generated in the GeneChip® Command Console® Software (AGCC) (Affymetrix, Santa Clara, CA, USA).

All samples are MIAME compliant and were handled according to SOP in the Microarray Center. A total of 27 CC and 19 MGC samples were amplified and hybridized to arrays. The 46 samples were submitted to ArrayExpress at EMBL using MIAMExpress. The experiment accession number is E-MTAB-4012.

### Formulation of Classifiers

Classifiers were constructed based on expression matrices obtained by Plier normalization [18]. Following the normalization, the data was initially filtered to include only probe sets with functional annotation (both protein coding and non-protein coding); reducing the number of probe sets included in the analysis to 28,054. The formulation and performance assessment of the classifier models was performed on this data set, with and without, an additional pre-filtering step to exclude probe sets expressed below back-ground level (average expression above 10). Back-ground filtering resulted in the inclusion of 17,279 probe sets in the CC data set and 15,146 probe sets in the GC data set, respectively.

Based on these data sets, three different classification algorithms (diagonal linear discriminant analysis (dLDA) [19,20], k-nearest neighbors (K-NN) [20,21] and support vector machines (SVM) with linear kernel [22,23] were generated and tested. In order to formulate and select the best model for classification of LB, the misclassification rate for each classifier was estimated using leave-one-out cross-validation (LOOCV), during which we applied uni-variate t-tests with a grit of p-values (p < 0.001, p < 0.005, p < 0.01, p < 0.05) for selection of the optimal number of probe sets to include in each model. The overall accuracy, as well as PPV and NPV were derived for both the cumulus and granulosa data separately. The output of these analyses is presented for the optimal classification models and model parameters (S2 and S3 Tables). The overall best performing SVM classifier, based on the back-ground filtered data set with a p-value cut-off of 0.01, resulting in a gene signature of 34 probe sets, representing 30 unique genes, was selected as the final model for further validation (Table 1, Cumulus and granulosa Classifiers: PLIER annot and PLIER annot above bg).

**Table 1:** Performance of cumulus and granulosa cell support vector machine (SVM).

**Cumulus Classifier PLIER annot**		**Classifier Model**	**# PS in signature**	**Accuray (%)**	**Accuracy class (%)**	**Sensitivity**	**Specificity**	**PPV**	**NPV**	**# PS**
LB	SVM linear		82	81	83 (10/ 12)	0.833	0.8	0.769	0.857	28054
NP	SVM linear		82	81	80 (12/15)	0.8	0.833	0.857	0.769	28054
**Cumulus Classifier PLIER annot above bg**	**Classifier Model**		**# PS in signature**	**Accuray (%)**	**Accuracy class (%)**	**Sensitivity**	**Specificity**	**PPV**	**NPV**	**# PS**
LB	SVM linear		34	81	83 (10/ 12)	0.833	0.8	0.769	0.857	17279
NP	SVM linear		34	81	80 (12/15)	0.8	0.833	0.857	0.769	17279
**Granulosa Classifier PLIER annot**	**Classifier Model**		**# PS in signature**	**Accuray (%)**	**Accuracy class (%)**	**Sensitivity**	**Specificity**	**PPV**	**NPV**	**# PS**
LB	SVM linear		376	11	20 (2/10)	0.2	0	0.182	0	28054
NP	SVM linear		376	11	0 (0/9)	0	0.2	0	0.182	28054
**Granulosa Classifier PLIER annot above bg**	**Classifier Model**		**# PS in signature**	**Accuray (%)**	**Accuracy class (%)**	**Sensitivity**	**Specificity**	**PPV**	**NPV**	**# PS**
LB	SVM linear		108	11	20 (2/10)	0.2	0	0.182	0	15146
NP	SVM linear		108	11	0 (0/9)	0	0.2	0	0.182	15146

The classifiers were trained to classify live birth (LB) versus no pregnancy (NP). The table shows the performance with and without pre-filtering for probe sets expressed above background level. The models CC and MGC PLIER annot were formulated using only annotated probe sets (both protein coding and non-protein coding) and the models PLIER annot above bg were based on only annotated probe sets with an average expression above 10 (unlogged.)

### Relative weight of the genes in the CC signature

Following the cross validation procedure, we calculated the cross-validation support for each gene showing the percentage of the cross-validation training sets in which each particular gene was selected. The cross-validation support shows the percentage of the cross-validation training sets in which each particular gene was selected, giving the relative strength (weight) of each gene in the signature.

### Predictive probability of LB classification

For the optimal CC classifier, the predictive probability (estimate of the probability of getting a particular class label) of each samples, was derived from the trained SVM model. This was achieved by transforming the SMV classifier into a probabilistic classifier by calculating the predictive probability of a sample being one or the other type using logit estimates as implemented in the e1071 R library [24]. The predictive probabilities of the final and optimal classifier are visualized in a scatter plot of the function, p(LB), live birth, by plotting the predictive probability on the y-axis for each consecutive sample shown on the x-axis.

### Statistical significance of the Error rate

A permutation test was performed in order to determine if the cross-validated misclassification rate of the final CC classification model was lower than expected by chance (Tusher et al. 2001; Simon et al. 2007). In 1000 random permutations of the class label, the entire cross-validation was repeated for classifying the random grouping of the samples. The proportion of the 1000 random permutations that resulted in a smaller or similar cross-validation misclassification rate as obtained with the real data determine the permutation p-value. The statistical significance of the error rate was determined both for the linear SVM, 1-NN, 3-NN and dLDA classifier.

### Gene function enrichment and Biological Networks

The cumulus gene signature was subjected to Ingenuity pathway analysis using IPA Ingenuity software Ingenuity®Systems (www.ingenuity.com). The list of the signature probe sets with p value and fold change was imported into Ingenuity. By application of the Ingenuity Pathway Knowledge Base each gene identifier was overlaid onto a global molecular network linking their functionality to the function of other genes and biological enriched functionality and mechanistic networks of these genes were then generated based on their connectivity and enrichment statistics. A network score was calculated based on the hyper-geometric distribution and calculated with the right-tailed Fisher’s exact test. The score is the negative log of this p-value. The score takes into account, the number of network eligible molecules in the network and its size, as well as the total number of network eligible molecules analyzed and the total number of molecules in the knowledge base that could potentially be included in the network. The score represents the chance of getting a network containing at least the same number of network eligible molecules by chance when randomly picking the number of genes that can be in networks from the knowledge base. The top molecular and cellular functions and networks are shown.

Downstream effect analysis was performed to predict the effect of directional gene expression change on biological processes based on the expected causal effects derived from the literature. The predicted effect is based on a value calculated by the IPA z-score algorithm. The z-score predicts the direction of change for the function. An absolute z-score of ≥ 2 is considered significant. A biological function is predicted to be Increased if the z-score is ≥ 2 and decreased if the z-score ≤ -2.

### External validation data set

The optimal CC classifier-signature of 34 probe sets (30 unique gene) was validated on an external data set generated on the Agilent platform using the Whole Human Genome Microarray 4x44K microarray [9]. The full data set comprising three subseries of CC microarray expression profiles was downloaded from the GEO repository at NCBI. The three samples sets used were part 1: GEO accession GSE37110, including 10 embryos of poor quality (EP) and 11 blastocyst (B); part 2: GEO accession GSE37117, including 18 EP and 18 blastocyst B, and part3: GEO accession GSE37116, including 13 EP and 11 B.

The microarray expression profiles were generated in a two-color array with dye-swap of replicate samples to avoid systematic noise in the data related to the choice of dye (cye-5 or cye-3). We downloaded the preprocessed data of normalized unlogged expression values representing one-color channel intensities. The data was used for validation of the gene signature after applying a log2 transformation. Gene symbols were used to select the probes on the 4x44K array that overlap with the unique genes in the 30-gene signature. Due to redundant representation of some of the genes on the Agilent array, the gene signature was represented by 68 redundant mRNA transcripts (representing 25 unique genes) on the Agilent 4x44 platform. For assessing the performance of this gene signature, represented by 25 unique genes, the probe with the intermediate expression value for each of the genes with redundant probes was used to define the 25-gene signature in the external data sets.

### Performance of signature on external data

The external data sub sets (GSE37110, GSE37116 and GSE37117) were tested one by one. For each sub set, a test set data object was constructed and the overlapping gene signature was tested using SVM classification with linear kernels. The predictive probabilities were derived and visualized along with the predictive probability of the training set samples.

### Receiver operating characteristic (ROC) curves

ROC curve characteristics analysis was performed on all data set (training and validation data) with the end point LB or NP in the training data, and blastocyst or embryo of poor quality used in the validation data set. A ROC curve was generated for each partition of the validation data set. The ROC curve was created by plotting the true positive rate (TPR, or sensitivity) versus the false positive rate (FPR, or 1—specificity). The area under the curve (AUC) indicates the degree of predictive ability of the gene expression, where 0.5 is random chance and 1.0 is perfect predictive ability. The ROC and AUC were produced using the functionality of the R library ROCR.

## Results

### Clinical presentation of patients in the study

A total of 46 expression profiles were generated, encompassing 27 CC samples and 19 MGC samples, of which 12 CC sample and 9 MGC samples, respectively, were obtained from follicles where the oocyte after implantation gave rise to a live birth of a healthy baby (LB) and 15 CC and 10 MGC samples, respectively, which did not lead to pregnancy (NP). The expression profiles of the CC and MGC samples were analyzed separately. The basic demographic parameters for the women which CC and MGC were analyzed are presented in S1 Table. No differences were present between the two groups.

### Construction of classifier for oocyte viability

We constructed a classifier to discriminate between patients where IVF treatment lead to live birth compared with no pregnancy, based on expression profiles on CC or MGC from the pre-ovulatory follicles.

We examined different methods for the construction of an optimal classifier of oocyte competence and tested several cut-off values of the test statistics with or with-out fold change and expression above back-ground filtering. Three different classifier models (diagonal linear discriminant analysis (dLDA), k-nearest neighbors and SVM with linear kernel function were generated and tested by LOOCV. Overall accuracy, as well as PPV and NPV were derived for both the cumulus and granulosa data separately, as shown in Table 1, S2 Table and S3 Table. A grit of p-values (p < 0.001, p < 0.005, p < 0.01, p < 0.05) was tested on all algorithms with LOOCV and the p-value cut-off (p < 0.01), which lead to the optimal performance was used to generate the gene signature of 34 probe sets, representing 30 unique genes which were used in the final classifier (S4 Table).

The overall best performance of the classifier models applied on the CC was achieved with PLIER normalized unlogged expression values by the linear SVM model and 3-NN and resulted in a LOOCV accuracy of 0.81 and 0.85, respectively (Table 1 and S2 Table). On the contrary, the dLDA algorithm showed low cross validation accuracy of 57% (S2 Table).

The performance range of the classifier models applied on PLIER normalized MGC with a p-value cut-off of 0.01 was 11% - 37% for dLDA, 3-NN (k = 3) linear SVM (S3 Table). Evaluation of the signature obtained using RMA normalized data showed slightly better results, although all analyses resulted in overall high error level and poor performance with no signature showing significant discriminative power to distinguish LB samples from NP (data not shown), showing that the low performance is probably independent of normalization strategy.

The CC data show high levels of variance between the samples within each group and relatively small overall expression differences between the LB and NP groups. To increase the power of the analysis we applied a filter to firstly exclude un-annotated probe sets and further exclude genes with an average expression below back-ground level (average expression below 10 across all CC samples) [25]. Training of the linear SVM classifier after the annotation filtering step, resulted in a signature of 82 probe sets with unambiguous annotation, 60 of which were annotated with gene symbol (protein coding) and 22 were annotated as other non-coding RNAs, hence excluding probe sets with unclear function or lacking annotation.

Rerunning the training of the of the linear SVM after filtering for genes expressed above background, the overall accuracy of the SVM with radial kernel was unchanged (81%), when using a signature representing 30 unique genes (Table 1). The full list of the 30 gene signature with p-value cut-off and fold-change is shown in S4 Table.

**Fig 1:**
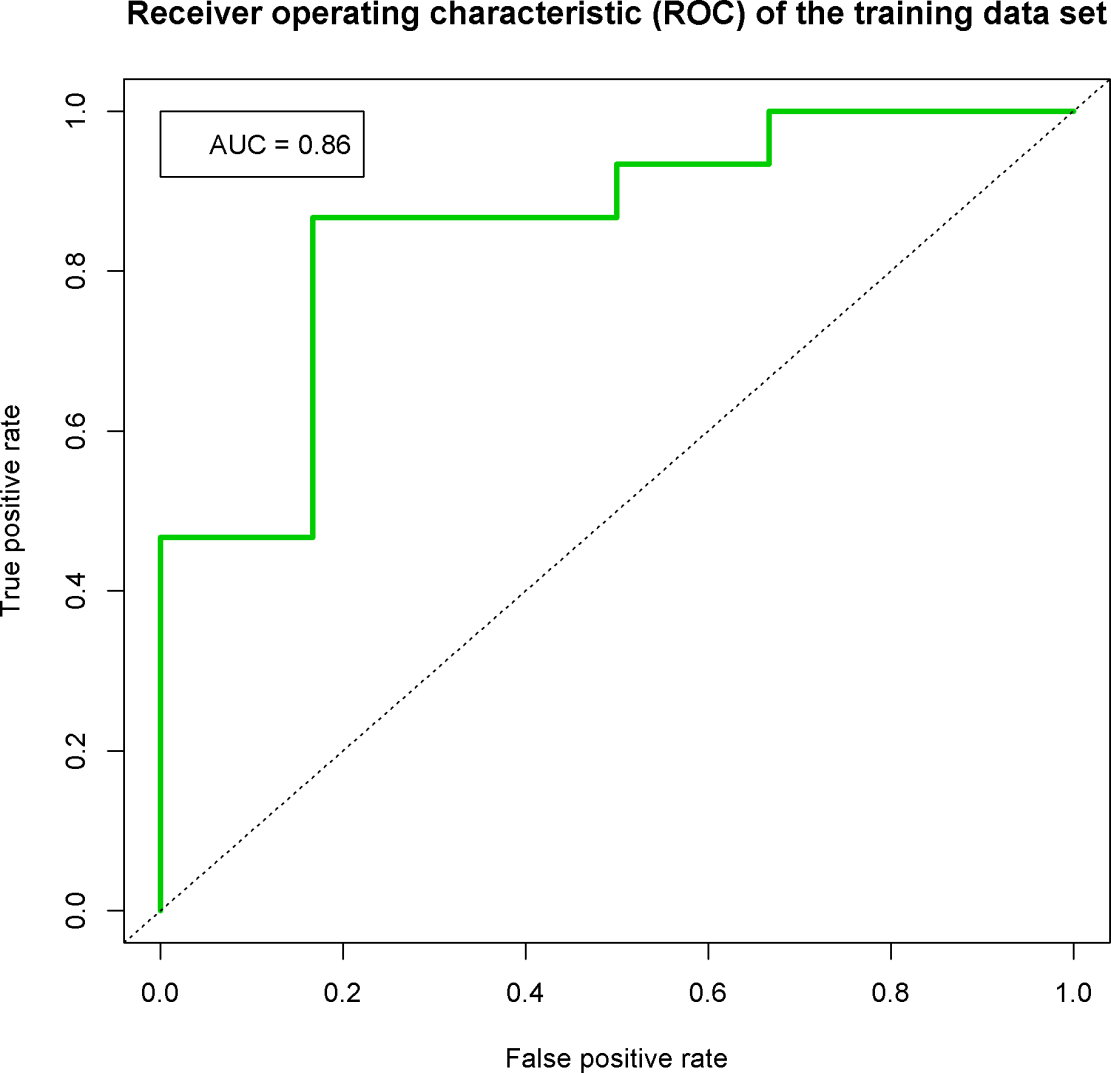
ROC curve of the training set. Receiver operating characteristic (ROC) curve for the binary classifier built to distinguish between live birth (LB) and no pregnancy (NP). The curve shows the true positive rate versus false positive rate, i.e. the tradeoff between sensitivity and specificity. The area under the curve (AUC), which captures the ability of the classifier to correctly group the patients with follicular adenoma and those with follicular carcinoma, is equal to 0.86. A perfect classifier will have an AUC of 1.0, whereas an AUC value of 0.5 indicates that the classification is random.

ROC curve analysis to distinguish between true positive rate and false positive rate of the final linear SVM classifier (annotated probes filtered to exclude probes expressed below background level) resulted in an AUC value of 0.86 indicating a good performance of the CC classifier (Fig 1).

**Fig 2:**
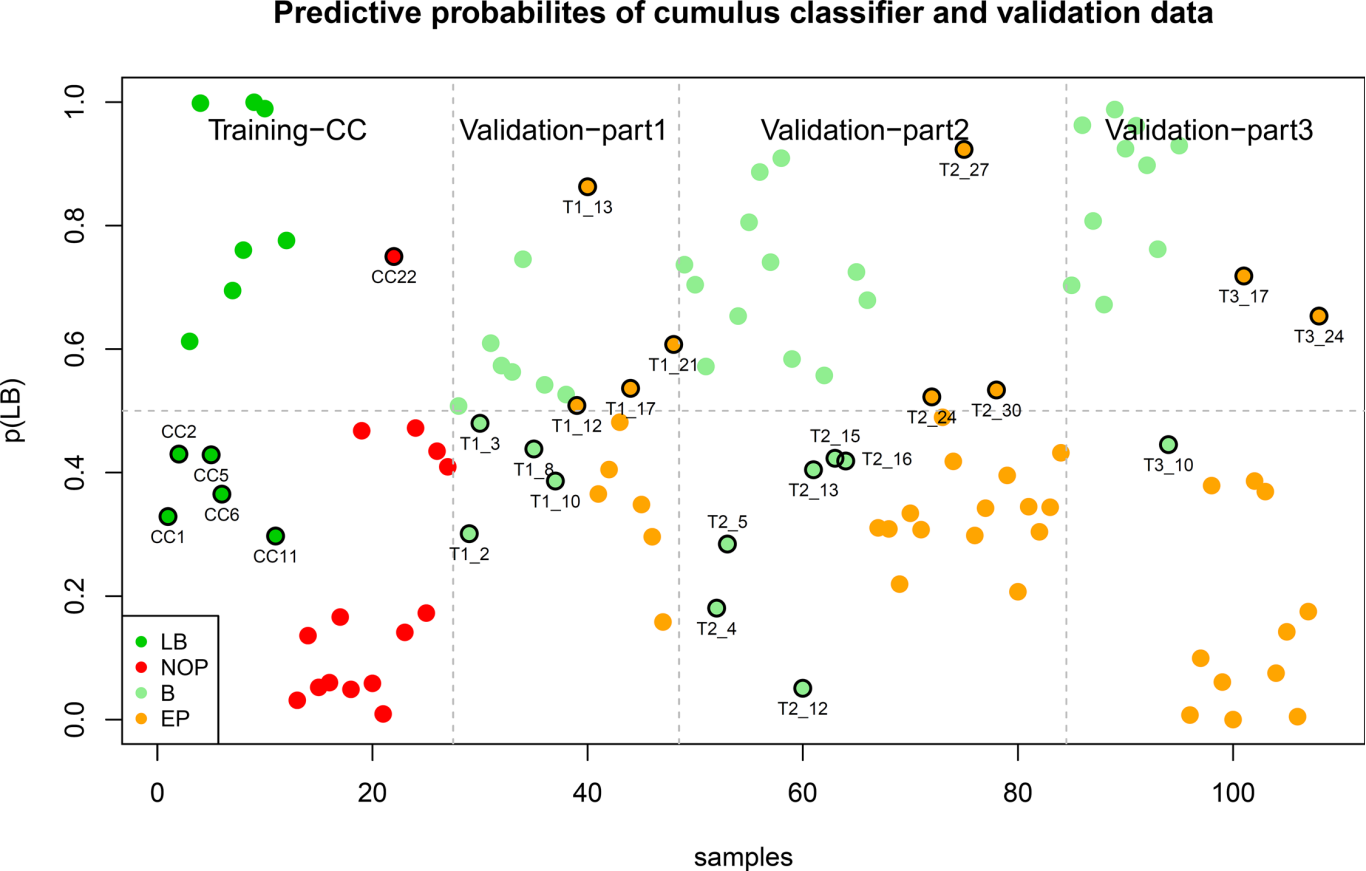
Predictive probability of cumulus training set samples and validation set samples. The predictive probability of the 30-gene signature is shown for the training set and after translation to the validation data set, part 1, 2 and 3, respectively. Each dot represents a sample and the color indicates the true (blinded) class. If a sample has a predictive value above 0.5 (p(LB) > 0.5), it is classified as predictive of leading to Live birth (LB) in the training set or reaching blastocyst (B) stage in the validation sub sets, validation-part1, validation-part2 and validation-part3, respectively. Samples with p(LB) below 0.5 are classified as predictive of no pregnancy (NP) in the training set and embryos of poor quality (EP) in the validation set. Samples which received an erroneously prediction according to their true class are indicated with a black circle and sample name.

We furthermore assessed the strength of each of the 30 genes in the signature by the cross-validation support, which shows the percentage of the cross-validation training sets in which each particular gene was selected (S4 Table). Analysis of the cross-validation support revealed that 12 genes had CV support of 100 percent; and more than half of the genes in the CC signature were included in 89 percent or more of the cross validation runs, hence receiving high weight in the classifier (see S4 Table). Visualization of the 30 gene signature by hierarchical clustering showed that samples with low expression of the top 100 percent CV support genes were the samples, which received low predictive probabilities of LB (Fig 2, S2 Fig).

In order to assess whether the misclassification rate was lower than what would be expected by chance we performed 1000 random permutation of the samples. For each permutation the entire LOOCV process was repeated and the proportion of the 1000 random permutations resulting in smaller misclassification rates determined the statistical significance of the classifiers error rate. The significance of the misclassification rate after 1000 permutations was 0.284, 0.002, 0.009, for the diagonal linear discriminant analysis classifier, the 3-nearest neighbors’ classifier and the linear SVM classifier, respectively. All but the d-LDA classifier was statistically significant compared to the random setting.

#### Predictive probabilities of the cumulus classifier

We used the trained SVM classifier to derive the predictive probability of a sample belonging to one class or the other by the use of logit estimates [24]. The predictive probability provides a likelihood estimate to the certainty of a sample to be classified as belonging to the predicted class of samples given the values of the genes in the signature. A sample is classified as being predictive of pregnancy and live birth if the predictive probability is above 0.5 and resulting in no ongoing pregnancy if the predictive probability is below 0.5 (Fig 2). A predictive probability of 0.5 reflects total uncertainty, whereas a probability of 1.0 is interpreted as complete certainty about the prediction. The accuracy of the 30 gene classifier estimated by LOOCV loops was 0.81 with 8/12 samples correctly predicted as LB and 14/15 samples classified as NP. Aside of the four LB sample, which received a wrong prediction by LOOCV, one other sample received a predictive probability below 0.5 showing five LB samples with low predictive probabilities compared to four being misclassified during LOOCV.

#### Validation of the cumulus gene signature on cross-platform external data

We tested the cumulus classifier on an external data set published by Feuerstein et al, 2012 downloaded from the public expression profile repository, gene expression omnibus (GEO) at NCBI [9]. The data set consisted of expression profiles of CCs originating from oocytes of IVF treated patients, which developed into blastocysts after 5/6 days of in-vitro culture or oocytes which were arrested at the embryo state. Three sub-sets of data were available as part of the study (part 1: GSE37110, part 2: GSE37116 and part 3: GSE37117) consisting of 21, 36 and 24 samples, respectively. Since these data were analyzed on the whole human genome oligo microarray 4x44K Agilent platform, the data sets were not directly comparable to the Affymetrix format. In order to test the CC signature of 30 unique genes, the gene signature was translated to the probe id of the Agilent platform using gene symbols, which resulted in a signature of 25 unique genes. The performance of this 25 gene signature was evaluated by linear SVM on pre-processed expression values downloaded from the GEO data base. Testing of the gene signature resulted in varying performance across the three parts of the data set, with part 3 data showing superior accuracy of 0.88 compared to 0.6 and 0.75 respectively for part 1 and part 2 (Table 2).

**Table 2:** Performance of the gene signature obtained during the formulation and training of the cumulus cell support vector machine on the external validation data set.

			Accuracy (%) of validation set, part 1			Accuracy (%) of validation set, part 2			Accuracy (%) of validation set, part 3		
Training set	# genes	Classification Model	Overall	LB	NP	Overall	LB	NP	Overall	LB	NP
PLIER above bg	25	SVM Linear, cost1	57	64	50	67	61	72	96	85	100
PLIER above bg	25	SVM linear, cost 2	**62**	64	60	**75**	67	83	**88**	91	85
PLIER above bg	25	SVM linear, cost 10	57	64	50	58	61	56	92	100	85

The table shows the classification accuracy of the binary classifier built to distinguish between live birth (LB) and no pregnancy (NP) on the three parts of the external cumulus expression data set (GEO accession: GSE37110, GSE37116 and GSE37117) using the linear support vector machine classifier with three settings of the cost parameter. Cost = 2 shows the best ability to classify the external data correctly.

The SVM predictions were translated into predictive probabilities and visualized alongside the predictive probabilities of the training data for comparison (Fig 2). In agreement with these results, ROC analysis resulted in AUC of 0.57, 0.65 and 0.88, respectively for the three sub data sets (S1 Fig).

#### Ingenuity pathway analysis

**Fig 3:**
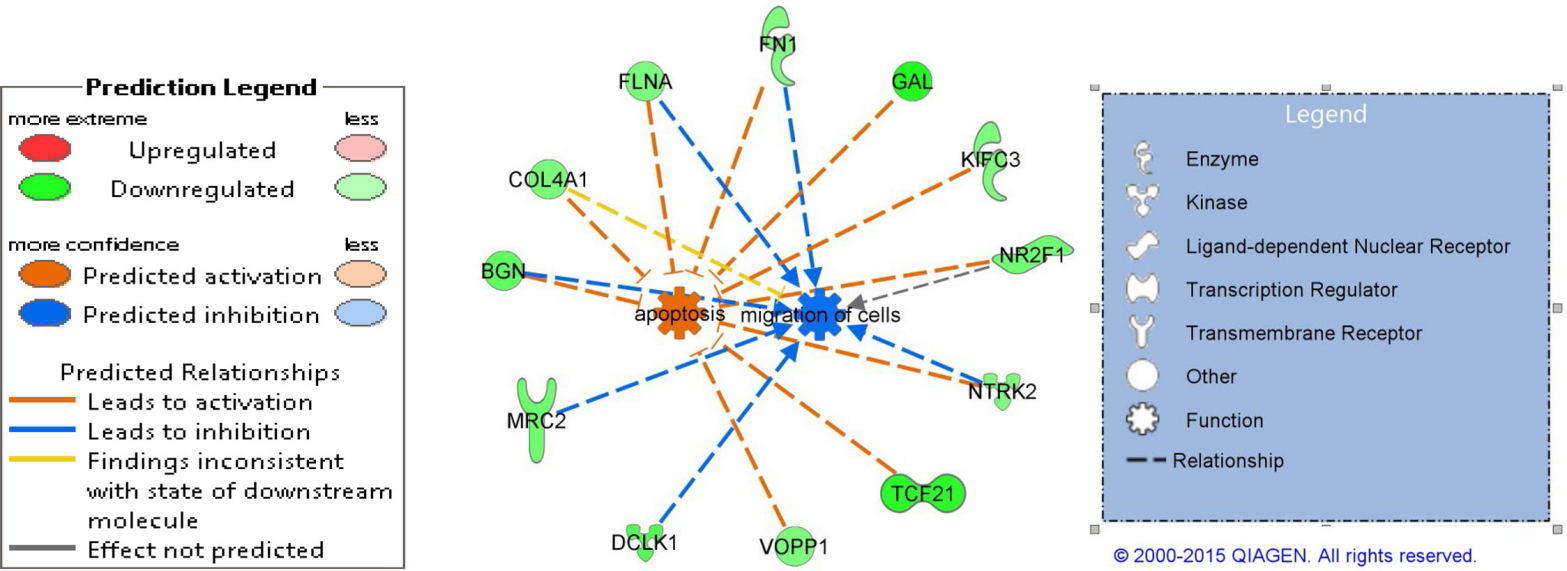
Predicted activation states. Ingenuity downstream effect analysis to predict the effect of directional gene expression resulted in two biological functions with significant activation z-scores indicative of predicted activation of apoptosis and decreased activity of cell migration. The mechanistic network of the implicated genes is shown along with the predicted relationship indicated by the color of the edges.

Despite the fact that gene classification signatures often can be difficult to interpret with respect to the biology of the underlying disease; and missing biologic interpretation does not warrant poor clinical usefulness of well-established biomarkers [26], the probe sets list including p-value, false discovery rate (FDR) q-value, and fold change of the comparison of LB versus NP, was imported into the Ingenuity Pathway Analysis (IPA) software to investigate biological functionality. Based on the relatively short gene signature list, no upstream activator molecules were detected. However, downstream effect analysis to predict the effect of directional gene expression change on biological processes based on the expected causal effects derived from the literature, resulted in two biological functions with significant activation z-scores. Down-regulation of 10 genes in the LB group collectively pointed to the activation of apoptosis with a z-score of 3.032 and an enrichment p-value of 0.018 (Fig 3). Four related bio functions in the categories of Cell Death and Survival, and Organismal Injury and Abnormalities were predicted to be activated in the LB group, although with non-significant z-scores below 2 (S5 Table). Six of the 10 genes were furthermore overlapping with a set of eight genes, which were predictive of decreased activity of cell migration (Fig 3, S5 Table). Additional four functional categories showed z-scores indicative of decreased activity although with low significance. These were microtubule dynamics, organization of cytoplasm, quantity of neurons and growth of neurites, all part of the biological categories Cellular Assembly and Organization, and Tissue Morphology (S5 Table).

**Fig 4:**
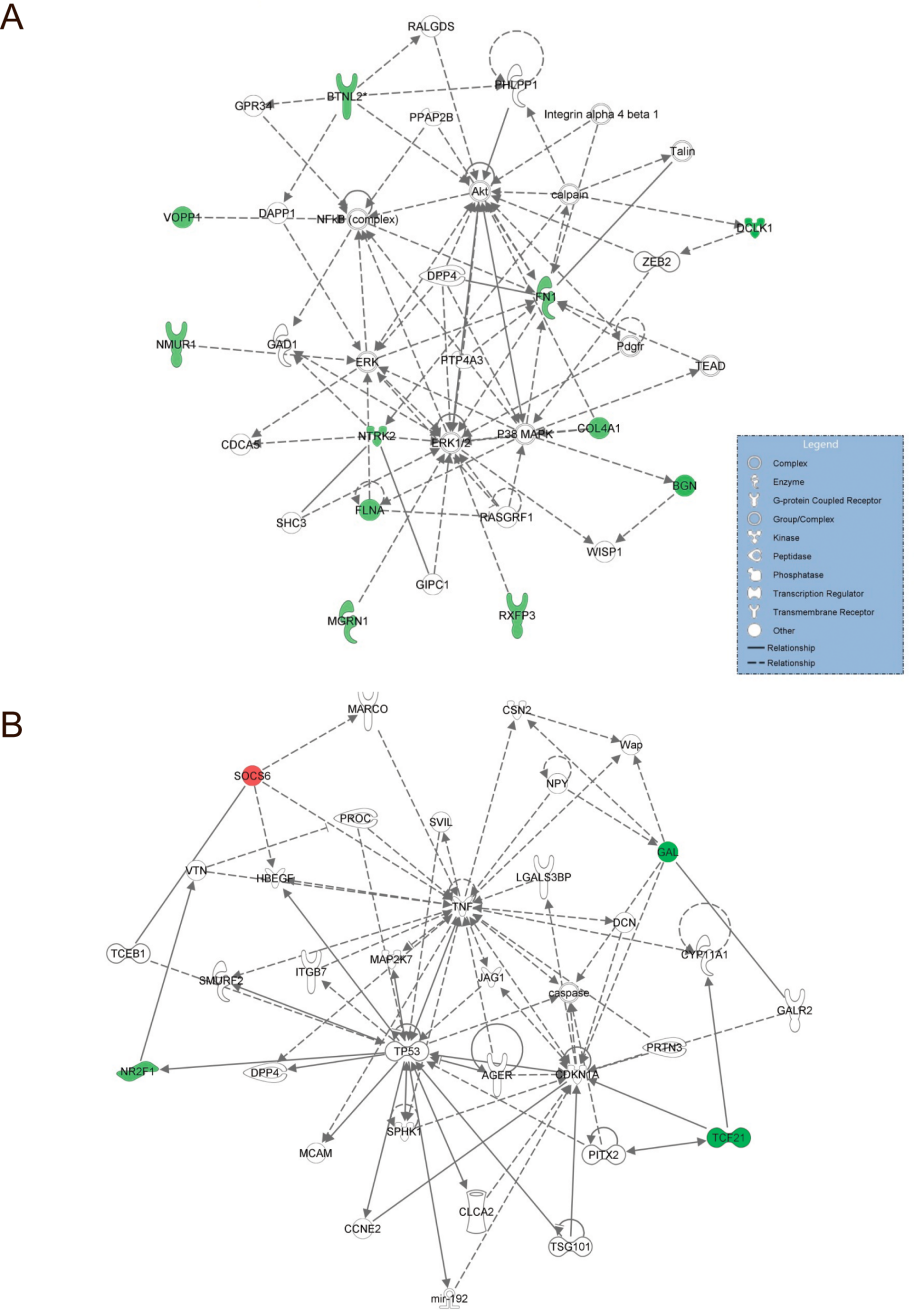
Mechanistic network. Mechanistic network based on the 30 probe set signature used to predict live birth in cumulus cells. (A) Top predicted functions represented by the network are Cellular Movement, Nervous System Development and Function, Cellular Growth and Proliferation. (B). Top predicted functions represented by the network are Cell Death and Survival, Cell-To-Cell Signaling and Interaction, Hematological System Development and Function.

Mechanistic networks were generated based on sets of connected upstream regulators that in conjunction would result in the gene expression changes observed in the dataset. Two mechanistic networks were generated with network scores above 6. The first network was enriched in functions related to Cellular Movement, Nervous System Development and Function, Cellular Growth and Proliferation with a network score of 24 (Fig 4A). Eleven of the 30 genes in the classifier gene signature are represented in the mechanistic network, all of which are down regulated in the LB group compared to NP. The second mechanistic network represents genes involved in Cell Death and Survival, Cell-To-Cell Signaling and Interaction, Hematological System Development and Function. Although only four of the 35 genes in the network are represented in the gene signature, they are part of the functional network involved in cell death, which is in line with the increased predicted activity of apoptosis (Fig 4B).

## Discussion

The study demonstrated that is possible to develop a classifier based on selection of 30 genes from whole genome microarray data from CC that predict the chance of pregnancy in an IVF setting with 81%. This classifier proved its value by prediction of the ability of embryos to reach the blastocyst stage in vitro from an independent data set. Therefore this classifier has the potential to be developed into a clinical useful tool if larger studies can confirm the results obtained in this study.

In contrast, we were not able to define a robust MGC classifier signature that could classify live birth with accuracy above random chance level.

The aim of this study was to use machine learning techniques to build CC and MGC transcriptomic classifiers to predict oocyte competence. Several combinations of microarray data pre-processing (RMA normalization) and feature selection were tested during cross validation rounds to derive the best performing algorithm and classifier gene signature [27–30].

Aside from the inherent heterogeneity of the data, we were able to correctly classify LB patients by leave one cross validation based on a 30 gene cumulus cell signature, and the cross validation support data showed that more than half of the genes in the signature were included in 89 percent or more of the cross validation runs, indicating that these genes have a stronger impact on the outcome and hence have a high relative weight in the classifier. We furthermore proved the classifier and the signature to be statistic significant compared to random classification of LB versus NP in 1000 random permutations of the samples as described [31].

The signature was furthermore tested for its ability to predict developmental competence as defined by the ability to reach the blastocyst stage in an external validation data set. The data represented 3 sets of transcriptomes from CC corresponding to oocytes that gave rise to 4-cell embryos on day 2 after fertilisation that either failed (incompetent) or succeeded to develop to full blastocysts (competent) after 5 days in vitro culture [9]. Our classifier gene signature succeeded in classifying these sets into competent and incompetent with 0.62, 0.75 and 0.88 accuracy (Table 2). The predictive power of the signature is increasing from part 1 through 3 of the data set, showing not only better performance (accuracy), but also more significant predictive power as assessed by increase in the significance of the predictive probability measure. It is unclear what the differences are between the three parts of the external data.

Whereas we observed a varying performance of the different CC classifiers depending on the choice of normalization and classification algorithm applied, all tested combinations of MGC classifiers showed poor performance in discriminating oocytes leading to live birth from oocytes which failed to establish a pregnancy. These data indicate that that gene expression in MGC does not reflect competence. However, the high degree of variability across samples, the small fold differences between the two end-points (NP and LB), combined with the lower number of samples in the MGC data set (19 MGC compared to 27 CC), with the resulting reduced power of the analysis may influence the ability to detect a set of discriminating signature genes during cross validation.

Further, explanation for the small expression changes observed in both cell types may be that the true signal of competence is blurred by the massive changes in the follicle originating from the ovulation trigger 34–36 hours before oocyte retrieval and isolation of cells [32–34]. Furthermore, it is possible that the true expression changes related to full developmental competence are masked due to the end-point of the analysis. The group of samples leading to no ongoing pregnancy may contain samples, which were obtained from oocytes which never passed the 8-cell stage nor reached the blastocyst stage, or reached the blastocyst stage, but failed to implant due to incompetence or a none receptive endometrium. Hence, they may express a transcriptome profile, which is more similar or different to the profile of the samples from the live birth group, depending on the time of which the development failed. Also, it has been established that the end-point in a classification study has a great impact on the performance of, and feasibility of formulating a classifier metric, which can in fact classify or predict a certain factor in a data set [35,36].

Since the overall aim of the project was to define predictors (genes) of LB and not to detect differentially expressed genes, a significance level of p < 0.01 in a univariate t-test was used for feature inclusion. This significance level is not sufficient significant to exclude false discoveries since no genes were significant after multiple testing correction (data not shown). This is in agreement with a recent study, where no genes were significantly expressed with FDR below 0.05 in CC and MGC of oocytes leading to pregnancy or not [13]. In some cases, better prediction is achieved by being more liberal about the gene sets included in the model although a biologically interpretation may not be meaningful [37].

Although many of the signature genes showed modest level of expression changes in the training data set and do not overlap with other studies [10–13,38,39]; they may indeed represent a biological blueprint of functionality related to competence [40,41]. This is one of the conclusions of a recent survey of hundreds of classification studies which showed that, despite of large dissimilarities between gene signatures depending on the choice of sample pre-processing and high level data analysis, the underlying biology of the different signatures in various classifiers which originated from the same data or examined the same biological question, was the similar [36,42].

The 30 annotated genes in the CC classifier signature were subjected to Ingenuity Pathway analysis. Downstream effect analysis suggests increased activity of apoptosis in the CC surrounding oocytes with developmental competence to develop, implant and succeed in birth of a healthy baby. None of the classifier signature genes have previously been shown to be regulated in CC of oocytes determined to be of good quality, mature or developmentally competent by different measures and standards [6,10–13,38,43]. Nonetheless, the underlying apoptotic activity has been investigated in follicular cells and it has been shown that a number of apoptotic follicular cells exist even in healthy follicles [44–46]. Studies using classical apoptotic markers (BCL2 and BAX expression, caspase activation, annexin V labeling and DNA fragmentation (TUNEL)) techniques to detect early and late stages of apoptosis, respectively, have shown a correlation between acquired developmental competence and a certain level of apoptosis [46–49] whereas several studies have suggested no or inverse correlation between apoptosis in CC and oocyte maturity and in vitro development after IVF/ICSI [47,50–53]. However evidence for correlation to pregnancy and birth has not been provided previously. An increase in apoptotic activity in CC connected to mature oocytes in MII as compared to GV stage has been suggested based on transcriptome comparisons [9]. Recent studies [51,52] show that increasing age of the women correlates to increased degree of apoptosis in CC. We have in the present study by the case control design aimed for minimizing a potential age induced bias in the results. The genes in the classifier which significantly enriches ‘increase activity of apoptosis’ in competent CC as compared to incompetent, are all reported in the literature to protect against apoptosis. If the level of apoptosis is related to competence remains to be answered, however, the expression level of central genes in apoptosis regulation, *P53*, *BAX*, *BCL-2* were not related to competence in our data.

Of the 30 genes in the classifier, 10 (*NR2F1*, *COL4A1*, *GAL*, *DCLK1*, *MRC2*, *FLNA*, *SOCS6*, *RAB33A*, *FN1*, *BGN*) showed an overlap with genes that previously have been shown to be differentially expressed between corresponding CC and MGC in the preovulatory follicle [54]. Interestingly, of these all but one (*COL4A1*) was 2–10 fold higher expressed in CC as compared to MGC indicating a specific function of the gene products in the CC compartment in the ovulatory follicle [54].

FN1, BGN, COL4A1 are all constituents of extracellular matrix and cell to cell adhesion involved in cumulus cell expansion in response to final maturation of the follicle [55–57]. A recent study of FSH induced superovulation in mice showed that increased amount of collagen type 4 (col4a1) in the cumulus of fully grown follicles after ovulation correlated with oocytes of lower developmental competence, as the superovulation conditions resulted in changed follicle morphology with delayed mucification and maturation [56]. Interestingly, we observed a higher expression of *COL4A1* in the CC of oocytes which did not result in an ongoing pregnancy, which might indicate a delay in maturation, which influence the developmental competence and promotes a delay in maturation that might lead to an asynchrony with downstream competence effects and early luteinization [56,58]. Furthermore, we observed that *FN1* was down regulated in CC of oocytes leading to ongoing pregnancy and live birth. *FN1* expression has been inversely linked to follicle maturation. Down regulation of this gene may indicate that matrix modelling is inactive, due to terminated follicular growth as a consequence of completed oocyte maturation [59].

In conclusion, we have analyzed the transcriptional profile of MGC and CC originating from individual follicles and developed a CC classifier, which showed a promising performance on external data on blastocyst development. This suggests that the gene signature at least partly include genes that relates to competence in developing to blastocyst. Further validation is needed on data representing implantation and birth to evaluate if the gene signature extends to classify not only blastocyst development as a major relevant marker of competence in IVF and ICSI, but ongoing pregnancy and live birth.

After completion of a large independent validation by a prospective randomized study comparing standard morphological evaluation with non-invasive classification of LB probability using CC expression profiling for embryo selection, we are aiming at establishing a web-server application allowing the public to perform CC expression profile classification (as exemplified in [23,60]).

## Supporting Information

S1 Fig ROC curve showing the true positive rate versus false positive rate of the validation data sets. (PDF)

S2 Fig Hierarchical cluster visualization of the 30 genes in the CC signature. (PDF)

S1 Table Patient demographics and baseline characteristics. (PDF)

S2 Table Performance of three different classification algorithms applied in cumulus microarray data analysis. (PDF)

S3 Table Performance of three different classification algorithms applied in granulosa microarray data analysis. (PDF)

S4 Table Gene Symbol and statistics of the 30 genes that constitute the cumulus cell classifier signature. (PDF)

S5 Table Functional enrichment and Predicted activation score. (PDF)
