# Supplementary material for: Competence Classification of Cumulus and Granulosa Cell Transcriptome in Embryos Matched by Morphology and Female Age
Source: PLoS One. 2016 Apr 29;11(4):e0153562. doi: 10.1371/journal.pone.0153562 (PMC4851390; doi:10.1371/journal.pone.0153562)
Supplement: S1 Fig — (PDF) [file pone.0153562.s001.pdf]

S1 Fig. ROC curve showing the true positive rate versus false positive rate of the validation data sets.

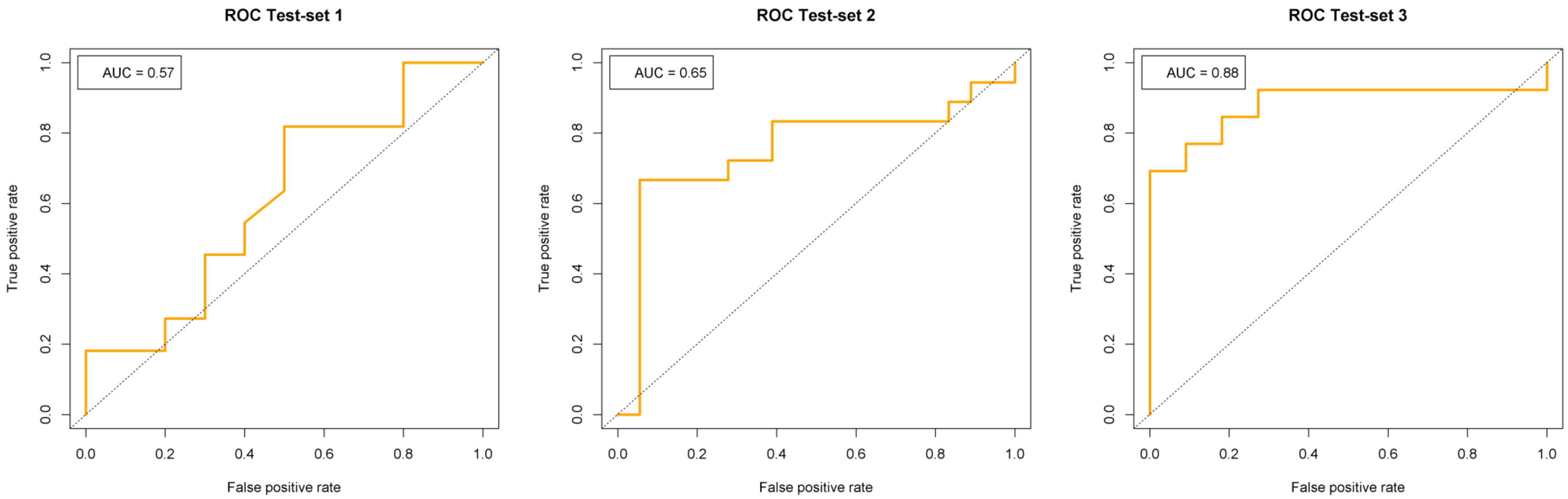

Receiver operating characteristic (ROC) curve for the binary classifiers ability to distinguish between oocytes that reached the blastocyst (B) stage and embryos of poor quality (EP) in the validation sub-sets. The curve shows the true positive rate versus false positive rate, i.e. the tradeoff between sensitivity and specificity. The area under the curve (AUC), which captures the ability of the classifier to correctly group the samples according to predicted live birth, is shown for each part of the validation data. The AUC of 1.0, whereas an AUC value of 0.5 indicates that the classification is random.
