# Supplementary material for: Competence Classification of Cumulus and Granulosa Cell Transcriptome in Embryos Matched by Morphology and Female Age
Source: PLoS One. 2016 Apr 29;11(4):e0153562. doi: 10.1371/journal.pone.0153562 (PMC4851390; doi:10.1371/journal.pone.0153562)
Supplement: S2 Fig — (PDF) [file pone.0153562.s002.pdf]

S2 Fig. Hierarchical cluster visualization of the 30 genes in the CC signature.

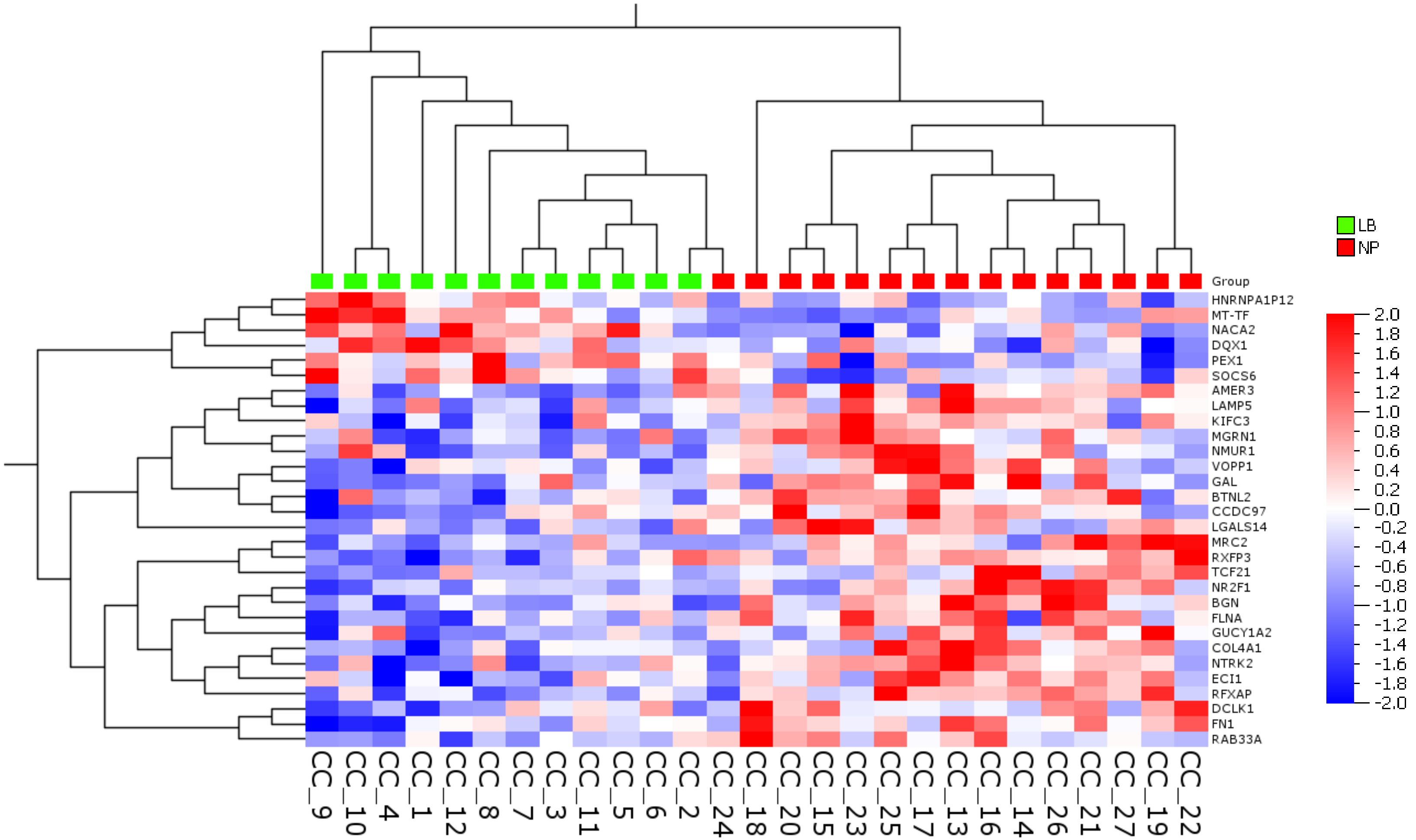

Relative expression level is indicated by pseudo colors where red is up regulated and blue is down regulated compared to the mean. The intensity of the color is determined by standard deviation from the mean as shown in the color bar to the right. LB samples are labeled by green and NP samples are labeled by red. The two-way clustering was performed using average linkage clustering and Euclidean distance.
