# Supplementary material for: Competence Classification of Cumulus and Granulosa Cell Transcriptome in Embryos Matched by Morphology and Female Age
Source: PLoS One. 2016 Apr 29;11(4):e0153562. doi: 10.1371/journal.pone.0153562 (PMC4851390; doi:10.1371/journal.pone.0153562)
Supplement: S1 Table — (PDF) [file pone.0153562.s003.pdf]

S1 Table. Patient demographics and baseline characteristics.

	Live Birth	No pregnancy	p-value
Age (years)[a]	31.9 ±2.9	33.1±2.4	0.22
Number of oocytes (no)[a]	10.1±5.2	10,8±4.7	0.72
Total FSH dose (IU)[a]	1538±150	1447±203	0.16
Treatment lenght (days)[a]	10.3±1.0	9.6±1.4	0.15
Primary cause of infertility[b]			
Female factor	7 (43.8)	8 (50.0)	0.92
Male factor	3 (18.3)	3 (18.3)	0.92
Unexplained	6 (37.5)	5 (31.3)	0.92

S1 Table. Patient demographics and baseline characteristics. Data are shown as mean ±SD or number (column percentage). $p<0.05$ is considered significant. [a]t-test. [b]chi-squared test .

S2 Table. Performance of three different classification algorithms applied during CC microarray data analysis.

Normalized data	Classification Model p < 0.01	Accuracy (%)	Accuracy / class (%)	Sensitivity	Specificity	PPV	NPV	# PS
**PLIER unlog LB**	LDA	59	33	0.417	0.733	0.556	0.611	82
**PLIER unlog NP**	LDA	59	73	0.733	0.417	0.611	0.556	82
**PLIER unlog LB**	3 NN	85	92	0.917	0.8	0.786	0.923	82
**PLIER unlog NP**	3 NN	85	80	0.8	0.917	0.923	0.786	82
**PLIER unlog LB**	SVM linear	81	83	0.833	0.8	0.769	0.857	82
**PLIER unlog NP**	SVM linear	81	80	0.8	0.833	0.857	0.769	82

Classification algorithms were trained on PLIER normalized data. Only annotated probe sets from the array were included in the analysis.
Performance parameters for each classification model were estimated using leave one out cross-validation.

S3 Table. Performance of three different classification algorithms applied during MGC microarray data analysis.

Normalized data	Classification Model p < 0.05	Accuracy (%)	Accuracy / class (%)	Sensitivity (%)	Specificity (%)	PPV (%)	NPV (%)	# PS
**PLIER unlog LB**	LDA	37	22	0.222	0.5	0.286	0.417	48
**PLIER unlog NP**	LDA	37	50	0.5	0.222	0.417	286	48
**PLIER unlog LB**	3 NN	11	11	0.111	0.1	0.1	0.111	48
**PLIER unlog NP**	3 NN	11	10	0.1	0.111	0.111	0.1	48
**PLIER unlog LB**	SVM linear	26	22	0.222	0.3	0.222	0.3	48
**PLIER unlog NP**	SVM linear	26	30	0.3	0.222	0.3	0.222	48

Classification algorithms were trained on PLIER normalized data. Only annotated probe sets from the array were included in the analysis.
Performance parameters for each classification model were estimated using leave one out cross-validation.

S1 Figure. ROC curve showing the true positive rate versus false positive rate of the validation data sets.

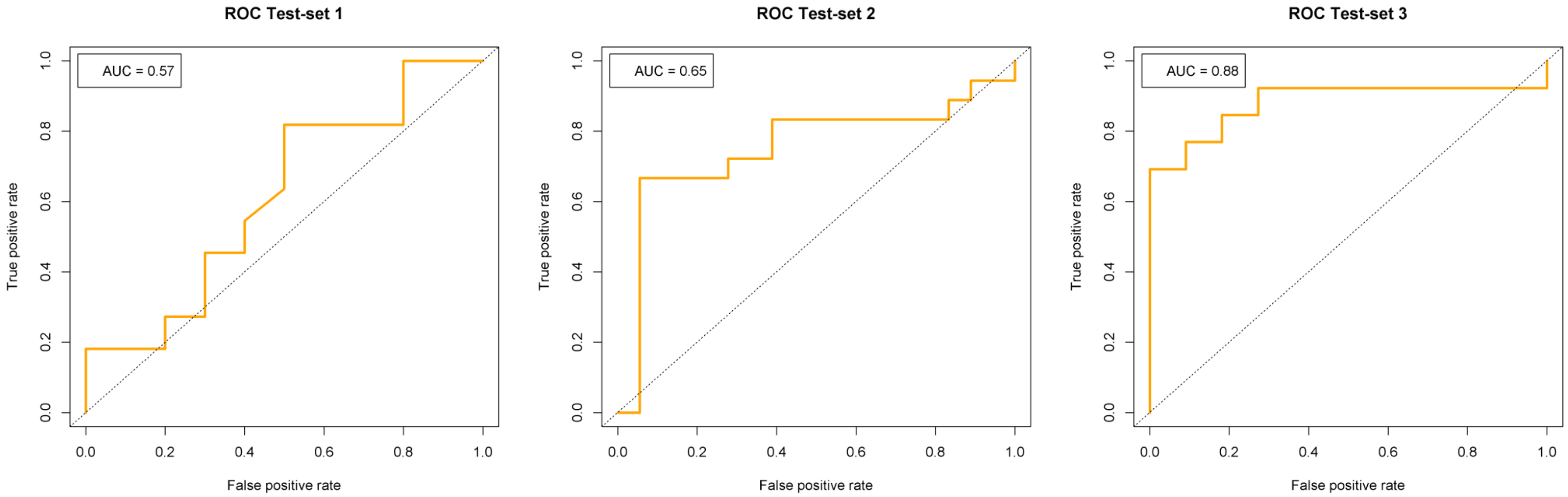

Receiver operating characteristic (ROC) curve for the binary classifiers ability to distinguish between oocytes that reached the blastocyst (B) stage and embryos of poor quality (EP) in the validation sub-sets. The curve shows the true positive rate versus false positive rate, i.e. the tradeoff between sensitivity and specificity. The area under the curve (AUC), which captures the ability of the classifier to correctly group the samples according to predicted live birth, is shown for each part of the validation data. The AUC of 1.0, whereas an AUC value of 0.5 indicates that the classification is random.

S4 Table. CC gene signature statistics.

Gene Symbol	Gene Title	P value	Mean LB	Mean NP	FC-sign	% CV-supp
GAL	galanin prepropeptide	0.005	107.68	237.26	-2.20	**100**
BGN	biglycan	0.001	41.03	63.72	-1.55	**100**
NR2F1	nuclear receptor subfamily 2, group F, member 1	0.002	35.02	48.65	-1.39	**100**
BTNL2	butyrophilin-like 2 (MHC class II associated)	0.002	9.99	12.90	-1.29	**100**
FLNA	filamin A, alpha	0.001	227.34	293.24	-1.29	**100**
RXFP3	relaxin/insulin-like family peptide receptor 3	0.001	10.75	13.69	-1.27	**100**
AMER3	APC membrane recruitment protein 3	0.001	14.39	18.04	-1.25	**100**
RFXAP	regulatory factor X-associated protein	0.003	10.20	12.65	-1.24	**100**
FN1	fibronectin 1	0.003	1841.19	2272.65	-1.23	**100**
HNRNPA1P12	heterogeneous nuclear ribonucleoprotein A1 pseudogene 12	0.005	208.09	177.08	1.18	**100**
NACA2	nascent polypeptide-associated complex alpha subunit 2	0.001	339.09	269.99	1.26	**100**
MT-TF	mitochondrially encoded tRNA phenylalanine	0.003	1719.58	1309.21	1.31	**100**
PEX1	peroxisomal biogenesis factor 1	0.005	21.93	16.83	1.30	**96**
DQX1	DEAQ box RNA-dependent ATPase 1	0.005	12.32	8.76	1.41	**93**
VOPP1	vesicular, overexpressed in cancer, prosurvival protein 1	0.005	29.58	37.88	-1.28	**89**
MGRN1	mahogunin, ring finger 1	0.005	56.26	68.25	-1.21	**89**
SOCS6	suppressor of cytokine signaling 6	0.005	23.85	18.61	1.28	**89**
ECI1	enoyl-CoA delta isomerase 1	0.006	26.84	33.44	-1.25	81
MRC2	mannose receptor, C type 2	0.006	23.08	32.97	-1.43	78
COL4A1	collagen, type IV, alpha 1	0.006	171.73	227.28	-1.32	67
GUCY1A2	guanylate cyclase 1, soluble, alpha 2	0.007	27.86	40.76	-1.46	59
NTRK2	neurotrophic tyrosine kinase, receptor, type 2	0.007	8.66	11.15	-1.29	56
DCLK1	doublecortin-like kinase 1	0.009	22.18	37.30	-1.68	48
RAB33A	RAB33A, member RAS oncogene family	0.008	13.13	22.02	-1.68	44
CCDC97	coiled-coil domain containing 97	0.009	31.93	38.34	-1.20	44
LAMP5	lysosomal-associated membrane protein family, member 5	0.008	12.07	16.48	-1.37	41
KIFC3	kinesin family member C3	0.008	12.48	15.77	-1.26	41
NMUR1	neuromedin U receptor 1	0.009	16.43	20.28	-1.23	41
TCF21	transcription factor 21	0.009	10.36	21.74	-2.10	37
LGALS14	lectin, galactoside-binding, soluble, 14	0.009	8.87	12.09	-1.36	33

Gene symbol and statistics for the 30 genes that constitute the cumulus cell classifier signature. FC-sign refers to the average directional fold change between the group of LB and NP patients. % CV-supp refers to the percentage of leave-one-out cross validation rounds where the particular gene was selected.

S2 Figure. Hierarchical cluster visualization of the 30 genes in the CC signature.

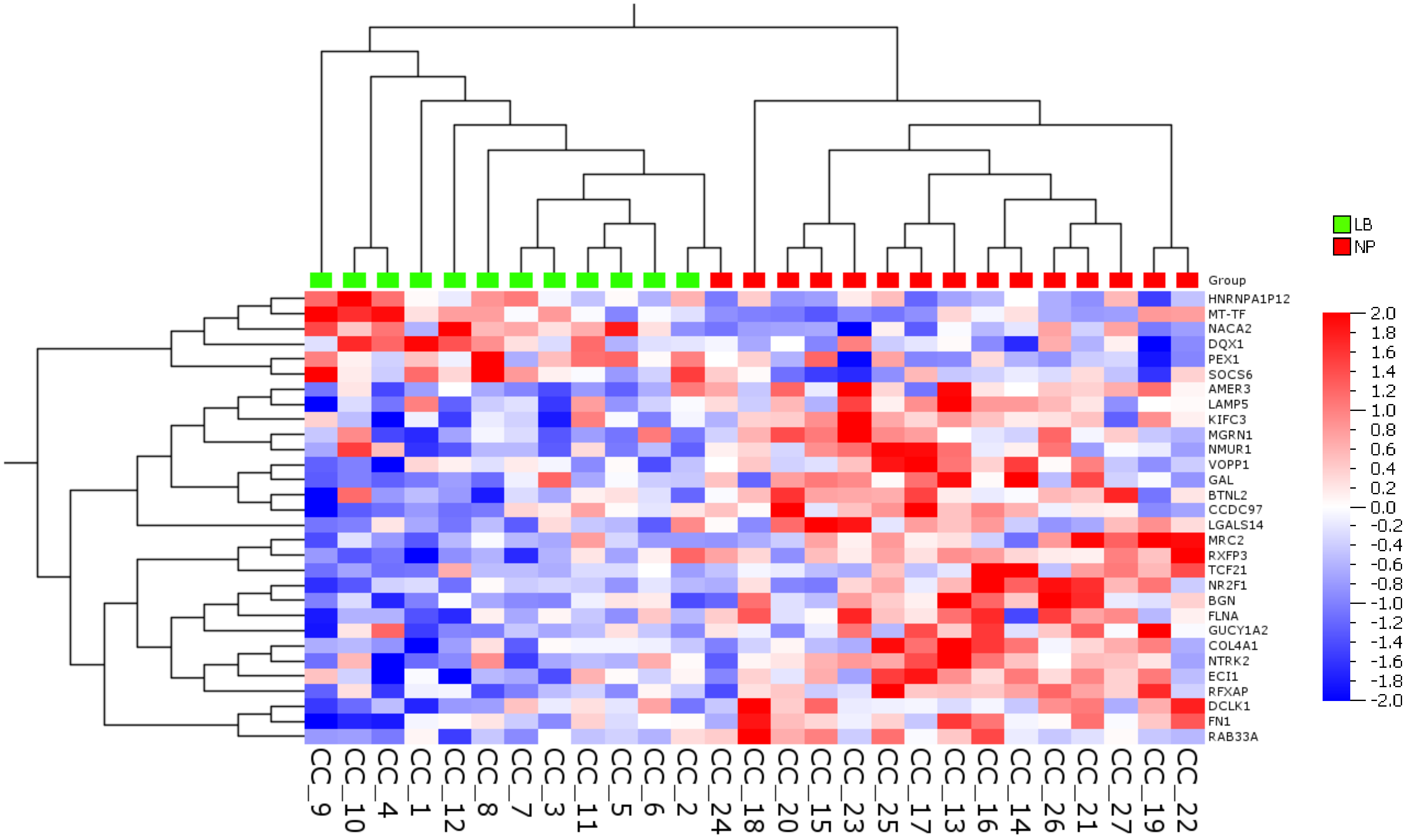

Hierarchical cluster visualization of the gene expression pattern of CC classifier signature. Relative expression level is indicated by pseudo colors where red is up regulated and blue is down regulated compared to the mean. The intensity of the color is determined by standard deviation from the mean as shown in the color bar to the right. LB samples are labeled by green and NP samples are labeled by red. The two-way clustering was performed using average linkage clustering and Euclidean distance.

Diseases or Functions Annotation	P value	Predicted Activation	Activation z-score	Molecules	# Molecules
**migration of cells**	**1.45E-02**	**Decreased**	**-2.012**	**BGN,COL4A1,DCLK1,FLNA,FN1,MRC2,NR2F1,NTRK2**	**8**
quantity of neurons	1.56E-04		-1.78	DCLK1,FLNA,GAL,NR2F1,NTRK2	5
organization of cytoplasm	5.11E-04		-1.534	DCLK1,FLNA,FN1,GAL,KIFC3,NR2F1,NTRK2,PEX1,RAB33A	9
microtubule dynamics	5.14E-04		-1.533	DCLK1,FLNA,FN1,GAL,KIFC3,NR2F1,NTRK2,RAB33A	8
growth of neurites	7.22E-04		-0.655	DCLK1,FN1,GAL,NTRK2,RAB33A	5
mass of organism	3.12E-03		0	GAL,NMUR1,NTRK2,SOCS6	4
apoptosis of tumor cell lines	2.79E-02		0.834	COL4A1,FN1,GAL,KIFC3,NTRK2,VOPP1	6
apoptosis of neurons	5.05E-03		1.938	FN1,GAL,NR2F1,NTRK2	4
Bleeding	8.89E-03		1.98	BGN,COL4A1,FLNA,FN1	4
Edema	3.73E-03		1.982	FLNA,FN1,GAL,NTRK2	4
**apoptosis**	**1.82E-02**	**Increased**	**3.032**	**BGN,COL4A1,FLNA,FN1,GAL,KIFC3,NR2F1,NTRK2,TCF21,VOPP1**	**10**

S5 Table. Functional enrichment and Predicted activation score. Biological functions with significant z-scores above 2 or below -2 are marked.
