# Supplementary material for: Competence Classification of Cumulus and Granulosa Cell Transcriptome in Embryos Matched by Morphology and Female Age
Source: PLoS One. 2016 Apr 29;11(4):e0153562. doi: 10.1371/journal.pone.0153562 (PMC4851390; doi:10.1371/journal.pone.0153562)
Supplement: S2 Table — (PDF) [file pone.0153562.s004.pdf]

**S2 Table. Performance of three different classification algorithms applied during CC microarray data analysis.**

| Normalized data | Classification Model p < 0.01 | Accuracy (%) | Accuracy / class (%) | Sensitivity | Specificity | PPV | NPV | # PS |
|---|---|---|---|---|---|---|---|---|
| **PLIER unlog LB** | LDA | 59 | 33 | 0.417 | 0.733 | 0.556 | 0.611 | 82 |
| **PLIER unlog NP** | LDA | 59 | 73 | 0.733 | 0.417 | 0.611 | 0.556 | 82 |
| **PLIER unlog LB** | 3 NN | 85 | 92 | 0.917 | 0.8 | 0.786 | 0.923 | 82 |
| **PLIER unlog NP** | 3 NN | 85 | 80 | 0.8 | 0.917 | 0.923 | 0.786 | 82 |
| **PLIER unlog LB** | SVM linear | 81 | 83 | 0.833 | 0.8 | 0.769 | 0.857 | 82 |
| **PLIER unlog NP** | SVM linear | 81 | 80 | 0.8 | 0.833 | 0.857 | 0.769 | 82 |

Classification algorithms were trained on PLIER normalized data. Only annotated probe sets from the array were included in the analysis.
Performance parameters for each classification model were estimated using leave one out cross-validation.
