# Supplementary material for: Competence Classification of Cumulus and Granulosa Cell Transcriptome in Embryos Matched by Morphology and Female Age
Source: PLoS One. 2016 Apr 29;11(4):e0153562. doi: 10.1371/journal.pone.0153562 (PMC4851390; doi:10.1371/journal.pone.0153562)
Supplement: S3 Table — (PDF) [file pone.0153562.s005.pdf]

S3 Table. Performance of three different classification algorithms applied during MGC microarray data analysis.

Normalized data	Classification Model $p < 0.05$	Accuracy (%)	Accuracy / class (%)	Sensitivity (%)	Specificity (%)	PPV (%)	NPV (%)	# PS
**PLIER unlog LB**	LDA	37	22	0.222	0.5	0.286	0.417	48
**PLIER unlog NP**	LDA	37	50	0.5	0.222	0.417	286	48
**PLIER unlog LB**	3 NN	11	11	0.111	0.1	0.1	0.111	48
**PLIER unlog NP**	3 NN	11	10	0.1	0.111	0.111	0.1	48
**PLIER unlog LB**	SVM linear	26	22	0.222	0.3	0.222	0.3	48
**PLIER unlog NP**	SVM linear	26	30	0.3	0.222	0.3	0.222	48

Classification algorithms were trained on PLIER normalized data. Only annotated probe sets from the array were included in the analysis.
Performance parameters for each classification model were estimated using leave one out cross-validation.
