# Supplementary material for: Competence Classification of Cumulus and Granulosa Cell Transcriptome in Embryos Matched by Morphology and Female Age
Source: PLoS One. 2016 Apr 29;11(4):e0153562. doi: 10.1371/journal.pone.0153562 (PMC4851390; doi:10.1371/journal.pone.0153562)
Supplement: S4 Table — (PDF) [file pone.0153562.s006.pdf]

S4 Table. CC gene signature statistics.

Gene Symbol	Gene Title	P value	Mean LB	Mean NP	FC-sign	% CV-supp
GAL	galanin prepropeptide	0.005	107.68	237.26	-2.20	**100**
BGN	biglycan	0.001	41.03	63.72	-1.55	**100**
NR2F1	nuclear receptor subfamily 2, group F, member 1	0.002	35.02	48.65	-1.39	**100**
BTNL2	butyrophilin-like 2 (MHC class II associated)	0.002	9.99	12.90	-1.29	**100**
FLNA	filamin A, alpha	0.001	227.34	293.24	-1.29	**100**
RXFP3	relaxin/insulin-like family peptide receptor 3	0.001	10.75	13.69	-1.27	**100**
AMER3	APC membrane recruitment protein 3	0.001	14.39	18.04	-1.25	**100**
RFXAP	regulatory factor X-associated protein	0.003	10.20	12.65	-1.24	**100**
FN1	fibronectin 1	0.003	1841.19	2272.65	-1.23	**100**
HNRNPA1P12	heterogeneous nuclear ribonucleoprotein A1 pseudogene 12	0.005	208.09	177.08	1.18	**100**
NACA2	nascent polypeptide-associated complex alpha subunit 2	0.001	339.09	269.99	1.26	**100**
MT-TF	mitochondrially encoded tRNA phenylalanine	0.003	1719.58	1309.21	1.31	**100**
PEX1	peroxisomal biogenesis factor 1	0.005	21.93	16.83	1.30	**96**
DQX1	DEAQ box RNA-dependent ATPase 1	0.005	12.32	8.76	1.41	**93**
VOPP1	vesicular, overexpressed in cancer, prosurvival protein 1	0.005	29.58	37.88	-1.28	**89**
MGRN1	mahogunin, ring finger 1	0.005	56.26	68.25	-1.21	**89**
SOCS6	suppressor of cytokine signaling 6	0.005	23.85	18.61	1.28	**89**
ECI1	enoyl-CoA delta isomerase 1	0.006	26.84	33.44	-1.25	81
MRC2	mannose receptor, C type 2	0.006	23.08	32.97	-1.43	78
COL4A1	collagen, type IV, alpha 1	0.006	171.73	227.28	-1.32	67
GUCY1A2	guanylate cyclase 1, soluble, alpha 2	0.007	27.86	40.76	-1.46	59
NTRK2	neurotrophic tyrosine kinase, receptor, type 2	0.007	8.66	11.15	-1.29	56
DCLK1	doublecortin-like kinase 1	0.009	22.18	37.30	-1.68	48
RAB33A	RAB33A, member RAS oncogene family	0.008	13.13	22.02	-1.68	44
CCDC97	coiled-coil domain containing 97	0.009	31.93	38.34	-1.20	44
LAMP5	lysosomal-associated membrane protein family, member 5	0.008	12.07	16.48	-1.37	41
KIFC3	kinesin family member C3	0.008	12.48	15.77	-1.26	41
NMUR1	neuromedin U receptor 1	0.009	16.43	20.28	-1.23	41
TCF21	transcription factor 21	0.009	10.36	21.74	-2.10	37
LGALS14	lectin, galactoside-binding, soluble, 14	0.009	8.87	12.09	-1.36	33

Gene symbol and statistics for the 30 genes that constitute the cumulus cell classifier signature. FC-sign refers to the average directional fold change between the group of LB and NP patients. % CV-supp refers to the percentage of leave-one-out cross validation rounds where the particular gene was selected.
