# Supplementary material for: Competence Classification of Cumulus and Granulosa Cell Transcriptome in Embryos Matched by Morphology and Female Age
Source: PLoS One. 2016 Apr 29;11(4):e0153562. doi: 10.1371/journal.pone.0153562 (PMC4851390; doi:10.1371/journal.pone.0153562)
Supplement: S5 Table — (PDF) [file pone.0153562.s007.pdf]

S5 Table. Functional enrichment and Predicted activation score.

Diseases or Functions Annotation	P value	Predicted Activation	Activation z-score	Molecules	# Molecules
**migration of cells**	**1.45E-02**	**Decreased**	**-2.012**	**BGN,COL4A1,DCLK1,FLNA,FN1,MRC2,NR2F1,NTRK2**	**8**
quantity of neurons	1.56E-04		-1.78	DCLK1,FLNA,GAL,NR2F1,NTRK2	5
organization of cytoplasm	5.11E-04		-1.534	DCLK1,FLNA,FN1,GAL,KIFC3,NR2F1,NTRK2,PEX1,RAB33A	9
microtubule dynamics	5.14E-04		-1.533	DCLK1,FLNA,FN1,GAL,KIFC3,NR2F1,NTRK2,RAB33A	8
growth of neurites	7.22E-04		-0.655	DCLK1,FN1,GAL,NTRK2,RAB33A	5
mass of organism	3.12E-03		0	GAL,NMUR1,NTRK2,SOCS6	4
apoptosis of tumor cell lines	2.79E-02		0.834	COL4A1,FN1,GAL,KIFC3,NTRK2,VOPP1	6
apoptosis of neurons	5.05E-03		1.938	FN1,GAL,NR2F1,NTRK2	4
Bleeding	8.89E-03		1.98	BGN,COL4A1,FLNA,FN1	4
Edema	3.73E-03		1.982	FLNA,FN1,GAL,NTRK2	4
**apoptosis**	**1.82E-02**	**Increased**	**3.032**	**BGN,COL4A1,FLNA,FN1,GAL,KIFC3,NR2F1,NTRK2,TCF21,VOPP1**	**10**

Biological functions with significant z-scores above 2 or below -2 are marked.
